# Co-regulation of biofilm formation and antimicrobial resistance in *Acinetobacter baumannii*: from mechanisms to therapeutic strategies

**DOI:** 10.1007/s10096-023-04677-8

**Published:** 2023-10-28

**Authors:** Sérgio G. Mendes, Sofia I. Combo, Thibault Allain, Sara Domingues, Andre G. Buret, Gabriela J. Da Silva

**Affiliations:** 1 Departments of Biological Sciences, Inflammation Research Network, University of Calgary, 2500 University Dr. N.W, Calgary, T2N 1N4 Canada; 2 Faculty of Pharmacy, University of Coimbra, 3000-548 Coimbra, Portugal; 3 Centre for Neuroscience and Cell Biology, University of Coimbra, 3000-548 Coimbra, Portugal

**Keywords:** *Acinetobacter baumannii*, Biofilm, Antimicrobial resistance, Co-infection, COVID-19, Quorum sensing

## Abstract

In recent years, multidrug-resistant *Acinetobacter baumannii* has emerged globally as a major threat to the healthcare system. It is now listed by the World Health Organization as a priority one for the need of new therapeutic agents. *A*. *baumannii* has the capacity to develop robust biofilms on biotic and abiotic surfaces. Biofilm development allows these bacteria to resist various environmental stressors, including antibiotics and lack of nutrients or water, which in turn allows the persistence of *A*. *baumannii* in the hospital environment and further outbreaks. Investigation into therapeutic alternatives that will act on both biofilm formation and antimicrobial resistance (AMR) is sorely needed. The aim of the present review is to critically discuss the various mechanisms by which AMR and biofilm formation may be co-regulated in *A*. *baumannii* in an attempt to shed light on paths towards novel therapeutic opportunities. After discussing the clinical importance of *A*. *baumannii*, this critical review highlights biofilm-formation genes that may be associated with the co-regulation of AMR. Particularly worthy of consideration are genes regulating the quorum sensing system AbaI/AbaR, AbOmpA (OmpA protein), Bap (biofilm-associated protein), the two-component regulatory system BfmRS, the PER-1 β-lactamase, EpsA, and PTK. Finally, this review discusses ongoing experimental therapeutic strategies to fight *A*. *baumannii* infections, namely vaccine development, quorum sensing interference, nanoparticles, metal ions, natural products, antimicrobial peptides, and phage therapy. A better understanding of the mechanisms that co-regulate biofilm formation and AMR will help identify new therapeutic targets, as combined approaches may confer synergistic benefits for effective and safer treatments.

## Introduction

The introduction, misuse, and overuse of antibiotics have facilitated the development of antimicrobial resistance (AMR) far beyond its original, natural, selection-based evolution [1, 2]. AMR occurs when microorganisms no longer respond to medicines they previously were sensitive to. AMR dissemination is facilitated by the horizontal transfer of a broad range of antibiotic resistance genes in bacteria from humans, animals, and in the environment [2–4]. Hence, a One-Health-based approach is key to our attempts at better understanding and, ultimately, solving this global threat [2, 5, 6]. As the clinical pipeline of new antimicrobials dries up, increased difficulties in treating bacterial infections due to AMR lay the terrifying foundations for emerging pandemic-size healthcare challenges [2, 7].

Bacteria may live as free-swimming, planktonic organisms. In their natural environments, however, bacteria are mostly sessile, adhered to a substrate, and form complex multispecies communities known as biofilms. Biofilms are the cause of enormous medical challenges and represent a life form that naturally resists exposure to environmental attacks, including that of antibiotics [8]. Biofilm communities secrete their own extracellular matrix, typically consisting of polysaccharides, proteins, and DNA. This matrix reduces the free diffusion of antimicrobials into the mature biofilms and facilitates the development of antibiotic resistance, which makes biofilms significantly more difficult to eradicate than planktonic organisms. Intense ongoing research initiatives investigate quorum sensing molecules, as well as the genes and second messengers, such as c-di-GMP and cAMP, that are implicated in biofilm formation [8–12]. The mechanisms whereby these genetic biofilm regulators may affect the development of AMR remain poorly understood. Research into co-regulatory mechanisms of biofilm and AMR development is sorely needed, as it may help identify new antimicrobial targets.

*Acinetobacter baumannii* is an opportunistic pathogen responsible for many nosocomial infections that include ventilator-associated pneumonia (VAP) and bloodstream infections, especially in patients hospitalized in intensive care units (ICUs) [13–15]. New risks have appeared with the emergence of the COVID-19 pandemic. The COVID-19 pandemic has spawned an overuse of antimicrobials, and co-infection with other respiratory pathogens in COVID-19 patients is an emerging concern. Even though the prevalence of secondary bacterial infections in COVID-19 patients may exceed 50%, reports about secondary infections or co-infections with opportunistic pathogens in COVID-19 patients still remain scarce [16–18]. Co-infections in COVID-19 patients may significantly worsen disease outcomes. Recent evidence suggests that co-infection and secondary infection with *A*. *baumannii* represent a significant threat to these patients. Indeed, infections with multidrug-resistant (MDR) *A*. *baumannii* in COVID-19 patients are being reported with increased frequency, particularly in ICUs [19–28]. Spread of MDR *A*. *baumannii* in hospitals can occur via several routes, including ventilator-associated transmission and air dispersal [23, 29, 30]. Recent findings demonstrate that the lower respiratory tract bacterial microbiome of critically ill COVID-19 patients favors the establishment of carbapenem-resistant *A*. *baumannii* [31, 32]. *A*. *baumannii* is known to form biofilms in host tissues, as well as on a variety of inert surfaces, including plastic and metals found in medical equipment [33–36]. This ability further complicates the control of such infections and warrants new prevention measures targeting biofilm-forming antimicrobial-resistant *A*. *baumannii* [13, 15].

With a focus on *A*. *baumannii*, the aim of this review is to discuss mechanisms that control biofilm formation and AMR development. We discuss the current understanding of genes that may co-regulate both of these processes as a path towards the development of novel therapeutic strategies.

## Clinical significance of *A*. *baumannii*

*A*. *baumannii* is a ubiquitous opportunistic pathogen belonging to the class of Gram-negative Gammaproteobacteria and is a non-fermentative, non-motile, catalase-positive coccobacillus [13, 36]. In addition to respiratory and bloodstream infections, *A*. *baumannii* may cause infections in the urinary tract and the skin and may lead to endocarditis and meningitis [13, 14, 30, 36, 37]. Some of these infections are related with the formation of biofilms, such as VAP and catheter-associated infections [37–39]. The mortality rate associated with infection by *A*. *baumannii* may exceed 50% [13, 30, 36, 40–44]. Recent reports also demonstrate that *A*. *baumannii* is responsible for community-acquired infections, such as community-acquired pneumonia with or without bacteremia. Often severe and fatal, these infections are more prevalent in patients with associated risks, such as diabetes mellitus and chronic lung diseases, and mortality can reach 64% [45–49].

*A*. *baumannii* expresses key virulence factors including lipopolysaccharides, capsular polysaccharides, proteases, phospholipases, outer membrane porins, outer membrane vesicles, biofilm-associated protein (Bap), iron-chelating systems, surface glycoconjugates, and protein secretion systems [14, 36, 50, 51]. However, the pathogenicity of the *Acinetobacter* genus, which today contains over 50 species, cannot be explained solely on the basis of phenotypic and chemotaxonomic methods, and virulence appears to be, at least in part, strain-dependent [14, 50, 51]. Indeed, this pathogen harbors a versatile genetic machinery that allows it to not only exhibit variable, strain-dependent virulence aspects but also rapidly generate environment-specific resistance and survival factors [14, 50–52]. As a result, it may be found in a broad range of habitats, including water, soil, food, and on the surfaces of medical equipment, and it may persist in environments that are inhospitable to many other bacterial pathogens. The most clinically relevant *Acinetobacter* spp. are grouped in the *Acinetobacter calcoaceticus-baumannii* (*Acb*) complex which includes 5 pathogenic *Acinetobacter* species, namely *A*. *baumannii*, *Acinetobacter nosocomialis*, *Acinetobacter pittii*, *Acinetobacter seifertii*, and *Acinetobacter dijkshoorniae*. A non-pathogenic one, *Acinetobacter calcoaceticus* also belongs to this group [14, 50–52]. *A*. *baumannii* exhibiting MDR profiles are encountered with increasing frequency [50, 52–56]. Resistance to last-resort antibiotics such as carbapenems and colistin has already been reported, allowing such strains to cause pan-drug-resistant infections that are presently impossible to eradicate [54–61]. A novel siderophore cephalosporin antibiotic, cefiderocol, was recently approved as a therapeutic agent for Gram-negative bacterial infections in both Europe and the USA [62]. As observed in *P*. *aeruginosa*, one of the innovative aspects of this antibiotic is that cefiderocol can cross the outer membrane, relying on a “Trojan horse” strategy. Cefiderocol creates chelating complexes with extracellular free iron, and thus, these complexes are transported into the bacteria via its iron transporters. Inside the cell, cefiderocol inhibits penicillin-binding protein 3, impairing cell wall synthesis [63–65]. Cefiderocol has proven to have potent activity against carbapenem-resistant *A*. *baumannii* [65–67]. However, cefiderocol-resistant *A*. *baumannii* isolates have already been reported [68–71]. It is estimated that approximately 44% of all *A*. *baumannii* isolates are MDR, with the highest incidence found in the Middle East and Latin America, where this rate may exceed 70%. In the European Region, the percentage of MDR *A*. *baumannii* can reach 43% [50, 72]. Results from the Central Asian and European Surveillance of Antimicrobial Resistance network and the European Antimicrobial Resistance Surveillance Network demonstrate an alarming increase in the reported cases of *Acinetobacter* spp., which have doubled (+121%), passing from 5375 cases reported for 2019 to 10885 cases reported for 2021. Regarding the percentage of carbapenem-resistant *Acinetobacter* spp., this value varied from below 1% to over 50% throughout the region. East and South Europe were the areas that showed the highest percentages of carbapenem-resistant *Acinetobacter* spp. Moreover, between 2017 and 2021, MDR *Acinetobacter* spp. increased from 32.1 to 36.8%. [73, 74]. As a result, *A*. *baumannii* has become a critical healthcare problem worldwide; this threat has received even greater attention due to the high prevalence of this bacterium in patients with COVID-19 [22, 23, 32].

*A*. *baumannii* is on the World Health Organization’s drug-resistant bacteria and antimicrobial resistance research priority list, and the Center for Diseases Control and Prevention has classified carbapenem-resistant *Acinetobacter* as an urgent threat to public health [75, 76]. The critical concerns around AMR in *A*. *baumannii* are compounded by the ability of this pathogen to form biofilms on biotic and abiotic surfaces. Within biofilms, bacteria exhibit limited metabolic activity, and their extracellular polysaccharide matrix shelters them from antibiotics and host immune factors. As a result, therapeutic options to treat MDR biofilm-forming *A*. *baumannii* have become ineffective.

## *A. baumannii* biofilms

**Fig. 1:**
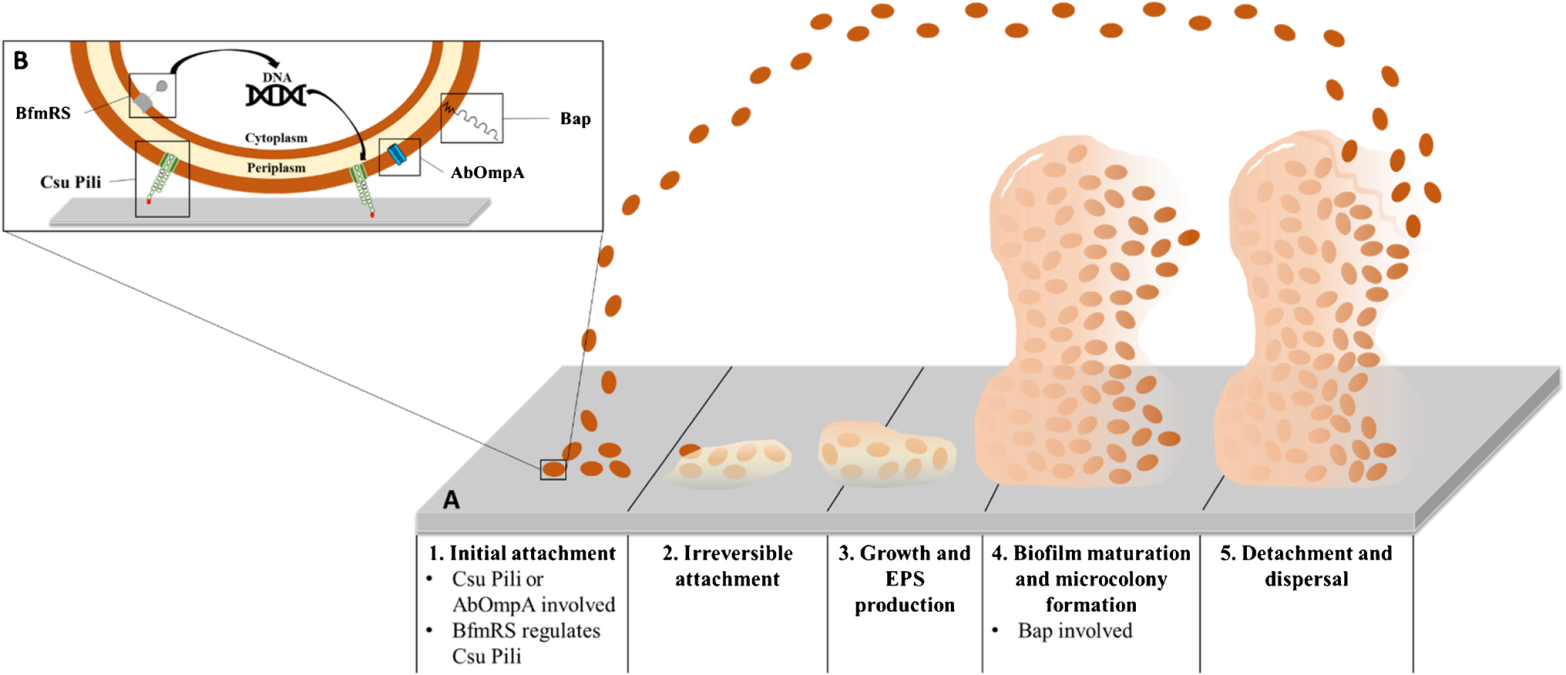
The five stages in biofilm formation (**A**) and *A*. *baumannii* virulence factors associated with biofilm formation (**B**). (**A**) Biofilm formation includes five different stages: (**1**) initial attachment, when planktonic cells reversibly attach to a surface; (**2**) irreversible attachment, when bacteria irreversibly attach to a surface and start cell-to-cell adhesion; (**3**) growth and extracellular polymeric substances (EPS) production, with bacteria starting to produce EPS; (**4**) biofilm maturation and microcolony formation, when biofilms become fully mature and microcolonies start to appear; and (**5**) detachment and dispersal, when biofilm bacteria start being released from the biofilm to make biofilms elsewhere. (**B**) *A*. *baumannii* has several virulence factors associated with biofilm formation, including the Csu Pili, which is related to the adhesion of the bacteria to abiotic surfaces such as plastic; AbOmpA, a porin involved in the adhesion of the bacteria to biotic surfaces such as epithelial cells; Bap, a protein involved in the maturation of biofilms and also BfmRS, a regulatory system involved in biofilms formation through the regulation of Csu Pili

*A*. *baumannii* rapidly forms sessile biofilm aggregates upon adherence to its substrate. Biofilm communities produce their own extracellular polysaccharide matrix and, in turn, release planktonic (free-swimming) bacteria that may establish new biofilms elsewhere, a bacterial survival process that exists throughout nature [8, 12, 77, 78] (see Fig. 1). The extracellular matrix contains polysaccharides, proteins, and nucleic acids and confers to the biofilm key viscoelastic, cohesive, and hydrating properties [8, 79, 80]. This dynamic mode of growth allows to retain water, to shelter against environmental stress, and facilitates quorum sensing and horizontal gene transfer [8, 80–83].

The formation of biofilms protects bacteria against antimicrobial substances, pH variability, UV radiation, extreme temperature, desiccation, nutrient starvation, and host immunity [81–84]. On abiotic surfaces, the biofilm mode of growth of *A*. *baumannii* protects against severe desiccation, which allows it to persist and may facilitate outbreaks [35, 36, 85–87]. Biofilms consume ambient nutrients, electron donors, and acceptors and may incorporate other bacterial species as well as dead and viable host cells [8, 78, 82, 83, 88–90]. The biofilm mode of growth may increase AMR by a given bacterial pathogen 100 to 1000-fold [87, 91–93]. A broad range of mechanisms cooperate to produce the elevated AMR observed in biofilms, including interactions of the antimicrobials with biofilm matrix elements, reduced bacterial growth rates, quorum sensing, and various drivers of antibiotic resistance that can fuel horizontal genetic transfer inside the biofilm [8, 90, 94–98]. Horizontal gene transfer is a phenomenon that occurs faster within biofilms than in planktonic cells. Inside biofilms, the bacterial evolution and development of drug-resistant bacteria can be achieved by the transfer of mobile genetic elements encoding antibiotic resistance genes, such as plasmids. This gene transfer can be fueled by the exposition to sub-minimum inhibitory concentrations (sub-MICs) of antibiotics inside the biofilm. Thus, biofilms are considered important reservoirs for the dissemination of AMR [95, 98, 99]. Several studies have established a positive correlation between biofilm formation and the degree of AMR in *A*. *baumannii*, with extensively drug-resistant (XDR) strains forming more robust biofilms than MDR strains [100–102]. Yet, other reports indicate that XDR strains tend to form weaker biofilms than non-MDR and MDR strains [93, 103], highlighting the urgent need to better understand co-regulatory mechanisms of biofilm formation and AMR.

## Quorum sensing

Quorum sensing is a cell-to-cell communication system that regulates bacterial behavior in both Gram-positive and Gram-negative bacteria in response to environmental stress [104–107]. This process depends on the production, detection, and down-stream signaling of secreted chemical molecules (autoinducers). Using this communication system, bacteria are capable to respond to increased cell density, to control biofilm growth, and to produce extracellular polysaccharides. It also allows bacteria to regulate genes implicated in virulence and drug resistance, an area of research receiving considerable attention in view of its potential to develop new therapeutics [108, 109]. A number of compounds and processes are known to contribute to quorum sensing (summarized in Table 1).

**Table 1:** Quorum sensing mechanisms regarding the autoinducer types in both Gram-positive and Gram-negative bacteria

Autoinducer type	Signaling molecule	Sensing mechanisms	Communication function type	Quorum sensing-related functions in representative specific bacteria		References
Autoinducer type I	*N*-acyl-homoserine lactones (AHL)	Two-component systems (TCS) involving synthase proteins and transcriptional regulators	Intraspecies communication in Gram-negative bacteria	*A*. *baumannii*	Biofilm formation, regulation of drug resistance genes, efflux pump-related genes and motility	[110–115]
				*P*. *aeruginosa*	Regulation and expression of virulence factors related genes; biofilm formation	[116–119]
	Oligopeptide autoinducers; small secreted peptides	TCS	Intraspecies communication in Gram-positive bacteria	Staphylococci	Biofilm formation and dynamics, regulation of virulence factors related genes	[120, 121]
Autoinducer type II	Furanosyl borate diester	TCS and internalization of AI-2	Interspecies communication in Gram-positive and Gram-negative bacteria	*Escherichia coli* and *Vibrio harveyi*	Regulation of expression of genes related to bioluminescence of *V*. *harveyi* and *lsr* locus regulation in *E*. *coli*	[122, 123]
Autoinducer type III	Threonine subproducts – 3,6-dimethylpyrazin-2-one	TCS QseBC	Interspecies communication – Enterohemorrhagic *E*. *coli*	Enterohemorrhagic *E*. *coli*	Related to *LEE* locus expression and Shiga toxin production in EHEC	[124–126]

In Gram-negative bacteria, quorum sensing compounds include *N*-acyl-homoserine lactones (AHL), composed of a homoserine lactone ring and a fatty acid acyl group variable in size, depending upon the bacterial species. Short (4 to 8 carbons) or long (10 to 16 carbons) AHL diffuses through the cell wall and acts as autoinducers [127–130]. Gram-positive bacteria use secreted oligopeptides as autoinducers and a two-component regulatory system (TCS) to regulate what genes and peptides need to be expressed. These TCS rely on membrane-bound histidine-kinase receptors and intracellular regulators [130, 131]. Furanosyl borate diester or tetrahydroxy furan (also called autoinducer-2, AI-2) is another type of signaling molecule used by both Gram-negative and Gram-positive bacteria and is known to serve as a signal for interspecies communications [132, 133]. Another autoinducer signal molecule involved in quorum sensing communication has been recently discovered in enterohemorrhagic *Escherichia coli* (EHEC) serotype O157:H7 and is referred to as autoinducer-3 (AI-3) [124]**.** AI-3 was characterized as 3,6-dimethylpyrazin-2-one [125]**.** EHEC bacteria sense AI-3 signaling molecules through the sensor kinase QseC belonging to the TCS QseBC (which is also involved in “sensing” of the host-derived hormones epinephrine and norepinephrine), thus enabling the control of expression of virulence genes, including those regulating the production of Shiga toxin [134].

Recent findings have significantly advanced our understanding of quorum sensing in regulating biofilm formation and AMR in both Gram-positive and Gram-negative bacteria, including in *A*. *baumannii* [50, 108–110, 135–138]. Recent evidence indicates that quorum sensing deficiency is associated with the formation of thinner biofilms that become susceptible to kanamycin [139]. In *P*. *aeruginosa*, quorum sensing regulates the expression of superoxide dismutase and catalase genes that confer resistance to hydrogen peroxide [140]. A three-day exposure of *A*. *baumannii* to sub-inhibitory concentrations of antibiotics increases biofilm formation and AMR in association with significant genome alterations linked to these phenotypic changes [141]. Pharmacological interference with the quorum sensing system reduces pathogenicity and facilitates the elimination of a given pathogen by host immunity [137, 142, 143]. These observations add to the ever-increasing number of reports supporting the hypothesis that biofilm formation and development of AMR are, at least in part, genetically co-regulated processes and make the inhibition of bacterial communication an attractive target for new drug development [98, 108, 109, 144–146]. Indeed, intensive ongoing research activities aim at developing quorum sensing inhibitors or quorum quenching enzymes to decrease the virulence of bacterial pathogens, including *A*. *baumannii* [138, 143, 147, 148].

**Fig. 2:**
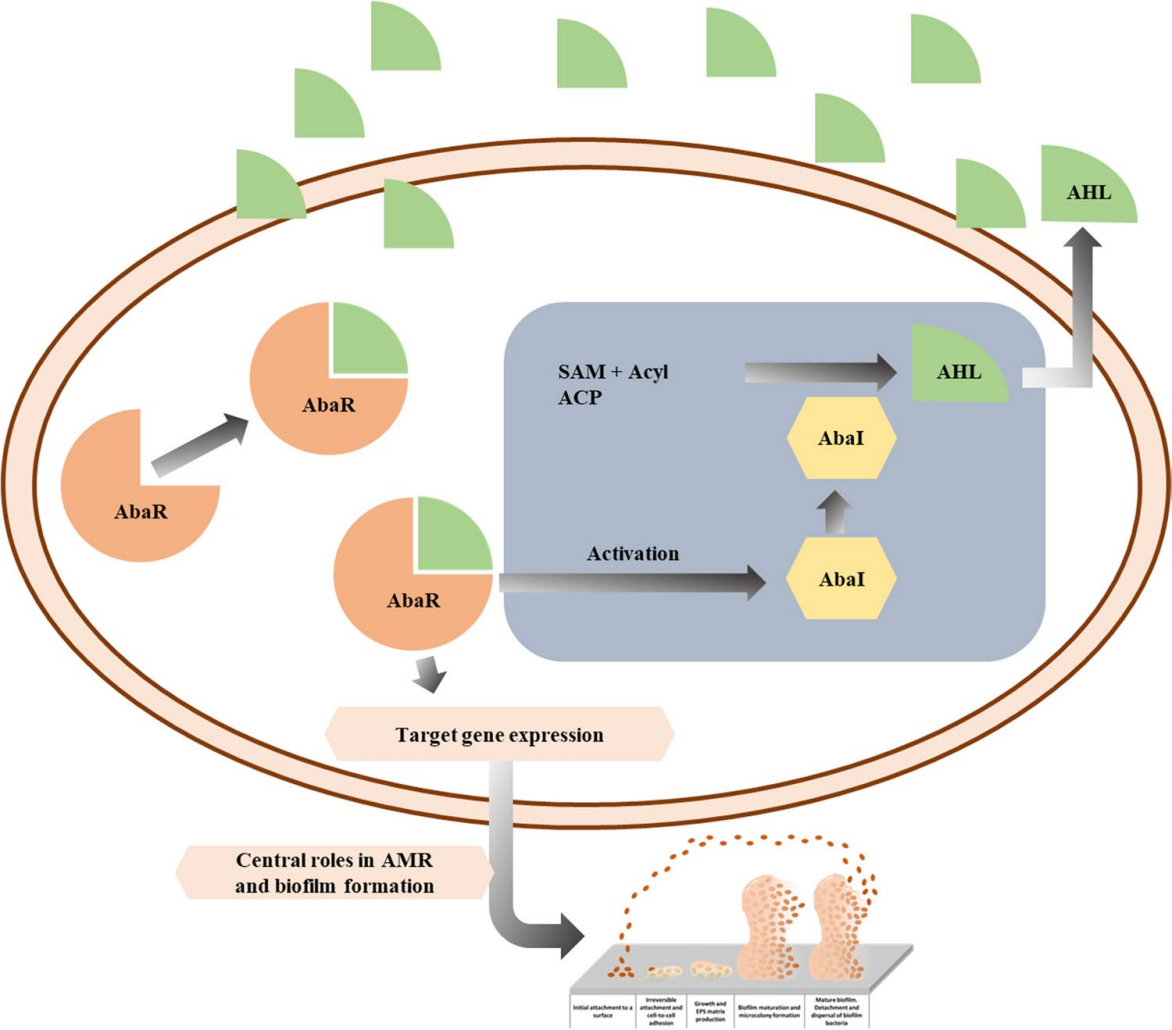
AbaI/AbaR quorum sensing system in *A*. *baumannii*. In this system, S-adenosyl methionine (SAM) and acyl-acyl carrier protein substrates (Acyl ACP) are the essential components for the production of AHLs. AbaI-regulated AHLs are released into the extracellular environment. Then, AHLs bind to the receptor AbaR present in the other cells, triggering a cascade of reactions that culminates in the control of expression of several target genes, including genes involved in biofilm formation and also for the production of more AHLs

Quorum sensing in *A*. *baumannii* is similar to what is observed in other Gram-negative bacteria, and it regulates biofilm formation, AMR, motility, and virulence [111, 112, 136, 137, 149–151]. AHL serves as the autoinducer-1. It is produced via the autoinducer synthase AbaI, which in turn binds to its cognate receptor AbaR (see Fig. 2), a system that is homologous to the canonical LuxI/LuxR system found in other Gram-negative bacteria [111, 149, 150]. AbaI-produced AHL binding to AbaR triggers a cascade of reactions leading to the quorum sensing response. *A*. *baumannii* AbaI is 27.5% identical and 47.3% similar to the LasI of a pathogenic and an environmental isolate of *P*. *aeruginosa*, respectively, and has a close resemblance to the LuxI family members described for *Vibrio fischeri* [110, 111, 138, 149]. *A*. *baumannii* also appears to express a *luxI* gene that contributes to AHL production [152]. In this bacterium, acyl side chains range from 10 to 16 carbons, although a number of *Acinetobacter* strains show various AHL profiles and produce more than one AHL [149, 153–155]. The most common AHL in *A*. *baumannii* is hydroxy-C12-homoserine lactone [110, 112, 155]. The quorum sensing system AbaI/AbaR is known to contribute to biofilm formation and antibiotic resistance in *A*. *baumannii* [110, 112]. Exogenous supplementation of AHL stimulates the formation of biofilms in non-biofilm-forming *A*. *baumannii* clinical isolates and enhances biofilm production in weakly adherent bacteria [156]. AHL also plays a role in *A*. *baumannii* drug resistance; an *abaI* deficient *A*. *baumannii* mutant was more susceptible to meropenem and piperacillin than the wild-type strain. However, its resistance was restored in the presence of AHL supplementation, promoting the expression of several drug resistance-related genes that include *bla*_OXA-51_, *ampC*, and the efflux pumps *adeA* and *adeB* genes [112]. Indeed, the AbaI/AbaR-dependent biofilm formation has been linked to overexpression of antimicrobial resistance genes, including those controlling efflux pumps [113, 114]. Yet, the molecular pathways regulating the complex AbaI/AbaR system remain incompletely understood.

## Biofilm-associated genes in *A*. *baumannii*

In *A*. *baumannii*, several genes are involved in biofilm formation and development. Some of these determinants and related functions are presented in Table 2.

**Table 2:** *A*. *baumannii* genes and their functions related to biofilm formation

Genes	Function	References
*csuA/BABCDE*	Type 1 chaperone-usher pili assembly system involved in the formation of the Csu pili, which plays a key role in adherence of *A*. *baumannii* bacteria to abiotic surfaces	[33, 86]
*abompA*	Codes for OmpA, a protein involved in the adherence of *A*. *baumannii* bacteria to biotic surfaces; plays a role in interaction with epithelial cells and biofilm formation	[157, 158]
*bap*	Involved in the adherence of *A*. *baumannii* bacteria to biotic surfaces, biofilm structure development and maturation, and water channel formation	[159, 160]
*bfmRS*	TCS involved in the regulation of the *csu* operon and, thus, regulation of biofilm formation	[161]
*adeRS*	TCS involved in the regulation of the resistance-nodulation-cell division (RND) efflux pumps superfamily-type AdeABC and in biofilm formation	[162, 163]
*pgaABCD*	Encodes for proteins involved in the synthetization of poly-β-(1-6)-N-acetylglucosamine, an important polysaccharide in biofilms	[164]
*abaI/AbaR*	Quorum sensing system in *A*. *baumannii*, being essential in biofilm development	[110, 115]
*epsA*	Encodes for a putative capsular polysaccharide export outer membrane protein (EpsA); it is required for capsule-positive phenotype, and it is associated with biofilm formation	[56, 165]
*ptk*	Encodes for a putative protein tyrosine kinase (PTK); it is required for capsule-positive phenotype, and it is associated with biofilm formation	[56, 101, 165]
*bla*_PER-1_	Encodes the PER-1 extended-spectrum β-lactamase, and it is associated with cell adherence and biofilm formation in *A*. *baumannii*	[100, 166–169]

Adherence is the critical step for the development of a biofilm. Thus, the factors that play a role in this first stage of biofilm formation, including Csu pili, AbOmpA, *A*. *baumannii* Bap, and the TCS BfmRS, will be discussed in the following paragraphs. Other genes that may also be involved in the co-regulation of biofilm formation in *A*. *baumannii* including *bla*_PER-1_, *epsA*, and *ptk* will be addressed.

### Csu pili

The attachment to a surface by planktonic bacteria is the first step in bacterial biofilm formation [170]. *A*. *baumannii* Csu pili contribute to the attachment and biofilm formation on abiotic surfaces [86]. In *A*. *baumannii*, Csu pili are formed by the type I chaperone-usher pili assembly system named CsuA/BABCDE. In these bacteria, the *csu* operon has six open reading frames, namely *csu* A/B, A, B, C, D, and E [86]. At the structure level, Csu pili are elaborated from four subunit proteins, CsuA/B, CsuA, CsuB, and CsuE, being assembled by CsuC and CsuD proteins [33, 86]. A positive correlation between biofilm formation and AMR, predominantly regulated by *csuE*, was reported recently. Concurrently, a high prevalence of *csuE* was observed among MDR *A*. *baumannii* clinical isolates, reaching 100% in some reports [56, 171]. At the Csu pili tip, CsuE is made of three hydrophobic finger-like structures responsible for the adherence of the bacteria to abiotic surfaces, such as hydrophobic plastics [33]. As a result, these bacteria exhibit a greater ability to form biofilms on polycarbonate hydrophobic materials than on glass, which is a hydrophilic material. The use of hydrophilic materials for medical devices may help prevent biofilm-associated infections [33, 35]. Sub-inhibitory concentrations of antibiotics like trimethoprim and sulfamethoxazole can inhibit *A*. *baumannii* ATCC 17978 biofilm formation. Through the inhibition of folate biosynthesis and then promoting folate stress, these antibiotics repress *csuA/B* expression, which, in turn, inhibits the development of biofilms [172]. *A*. *baumannii* Csu pili are not implicated in adherence to biotic surfaces, such as respiratory tract epithelial cells [173].

### *A*. *baumannii* outer membrane protein A (AbOmpA)

The OmpA of *A*. *baumannii*, also known as OmpAb [174], Omp38 [175, 176], or AbOmpA [177] (hereafter AbOmpA), is a permeability-relevant porin protein present in the outer membrane (OM) [174]. AbOmpA is considered as one of the major proteins in the OM of *A*. *baumannii*, and it is involved in specific processes of pathogenesis, such as adherence to and invasion of the host epithelial cells [157, 158, 174], disruption of the mitochondria, and cell death [176, 177]. AbOmpA is also related to the virulence capacity of outer membrane vesicles released from *A*. *baumannii* [178, 179]. AbOmpA may also induce dendritic cell death that can lead to a deficient host immune response [177]. In contrast, other studies have shown that AbOmpA stimulates the host immune system, activating and maturing dendritic cells and promoting the differentiation of CD4+ T cells [180]. Overexpression of AbOmpA presents a risk factor for the development of *A*. *baumannii* pneumonia and bacteremia, dissemination to organs like the lungs and spleen, and mortality [179, 181].

AbOmpA plays a role in *A*. *baumannii* biofilm formation in abiotic surfaces like polystyrene, and it is essential for the adherence of the bacteria to several biotic surfaces, such as human alveolar epithelial cells [158]. AbOmpA is also involved in intrinsic resistance to some antimicrobials. An *abompA* mutant *A*. *baumannii* ATCC 17978 strain showed increased susceptibility to trimethoprim than the wild-type strain. This increase in susceptibility may reflect the interaction of the OmpA-like domain of the AbOmpA with the inner membrane transporters of the resistance–nodulation–division efflux pumps superfamily [182].

### *A*. *baumannii* biofilm-associated protein (Bap)

Biofilm-associated proteins (Bap) have been identified in both Gram-positive and Gram-negative bacteria. These proteins share common structural and functional characteristics. They are bacterial surface proteins with a high molecular weight, possess a core domain of tandem repeats, and are involved in biofilm development [183].

First identified in *S*. *aureus* in 2001 [184], Bap was shown to be a cell wall protein associated with *S*. *aureus* primary attachment to abiotic surfaces, such as polystyrene, cell-to-cell adhesion, and, thus, biofilm formation. A *S*. *aureus* Bap homologous protein was then discovered in *A*. *baumannii* [159].

The *A*. *baumannii* Bap protein is involved in biofilm formation and maturation and participates in intercellular adhesion [159]. Bap protein plays an important role in bacterial adherence to human bronchial epithelial cells and human neonatal keratinocytes [160]. Also, Bap expression in *A*. *baumannii* is related to mature biofilm formation on abiotic surfaces, such as polypropylene and titanium. This protein is involved in the maintenance of cell surface hydrophobicity [160], a feature that is considered an important factor in adherence ability and biofilm formation in a variety of bacteria [185–187].

Many clinical *A*. *baumannii* strains have the *bap* gene [188, 189]. Bacteria that express the *bap* gene produce stronger biofilms, and the addition of affinity-purified Bap-specific antibodies inhibits biofilm formation [188]. In contrast, Bap-negative isolates recovered from bloodstream infections show a low biofilm formation ability [189]. The *bap* gene is often present in MDR *A*. *baumannii* strains, providing support to the hypothesis that this gene may help co-regulate biofilm formation and AMR in *A*. *baumannii* [190].

### The two-component regulatory system BfmRS in *A. baumannii*

TCSs are involved in the sensing and transduction of extracellular stimuli and are prototypically composed of a histidine kinase sensor and a response regulator. Histidine kinase senses a stimulus and sends the information to the response regulator by transferring a phosphoryl group, promoting conformational changes in the regulatory domain. In turn, the response regulator acts as an activator or a repressor for the specific gene’s transcription [191].

TCSs have been found in both Gram-positive and Gram-negative bacteria [161, 191, 192]. They play a role in several bacterial functions, including in the control of genes regulating efflux pumps [162, 193, 194] and biofilm formation [161, 195]. In *A*. *baumannii*, the TCS BfmRS regulates biofilm formation [161], capsular polysaccharide biosynthesis, as well as osmotic and oxidative stress responses. BfmRS may also modulate *A*. *baumannii* motility by controlling the expression of type IV pili [196, 197].

The BfmRS in *A*. *baumannii* is a TCS composed of a cytoplasmic response regulator, the BfmR, encoded by the *bfmR* gene, and a cytoplasmic membrane sensor kinase, the BfmS, which is encoded by the *bfmS* gene. The cytoplasmic protein BfmR is involved in biofilm development, cell morphology, and pellicle formation [161, 198]. BfmR is essential for attachment and biofilm formation in abiotic surfaces, such as polystyrene, as shown through a mutation in the *bfmR* gene. As a transcriptional activator, BfmR is involved in the regulation of Csu pili expression and, thus, adherence and biofilm development [161, 197]. Additionally, another TCS is also involved in the regulation of Csu pili, the TCS GacSC, and thus implicated in biofilm formation [199]. *A*. *baumannii* clinical isolates often express *bfmS* in association with elevated biofilm formation [56].

The BfmRS TCS is also involved in AMR. By mechanisms that are not yet fully understood, the response regulator BfmR is considered an important factor in AMR, including resistance to meropenem and polymyxin E concurrently with biofilm formation. BfmR is also involved in resistance to complement [200, 201]. Moreover, BfmR also participates in the regulation of *A*. *baumannii* tolerance to desiccation, explaining its persistence in hospital settings [202]. The sensor kinase BfmS is involved in *A*. *baumannii* biofilm formation. Mutation of *bfmS* also interferes with AbOmpA regulatory pathways and virulence [203, 204].

The *A*. *baumannii* BfmRS system is strongly associated with virulence and in the transcriptional regulation of the *k* locus, which is responsible for exopolysaccharide production in the presence of sub-MICs of antibiotics such as chloramphenicol and erythromycin [205, 206]. Furthermore, BfmRS is involved in resistance against β-lactams, both β-lactamase-dependent and β-lactamase-independent manner. In the presence of β-lactams aggression, BfmRS can regulate the production of β-lactamases and also control the cell division and cell wall degradation processes [207]. The fact that BfmRS is involved in AMR as well as in the regulation of biofilm formation through the control of Csu pili points to a role for this gene in the co-regulation of biofilm formation and AMR in *A*. *baumannii* [207].

### *A*. *baumannii bla*_PER-1_, *epsA*, and *ptk* genes

Other genes that may be involved in the co-regulation of biofilm formation and AMR in *A*. *baumannii* are *bla*_PER-1_, *epsA*, and *ptk*.

The *bla*_PER-1_ gene encodes for an extended-spectrum β-lactamase, the PER-1 [166–168]. *A*. *baumannii* that express *bla*_PER-1_ are associated with poor clinical outcome [167]. This gene appears to be essential in the adhesion capacity of MDR *A*. *baumannii* clinical isolates to respiratory epithelial cells and was found to contribute to biofilm formation on abiotic surfaces [208]. More research needs to assess whether this property may at least in part contribute to the poor outcome of patients infected with *A*. *baumannii* carrying the *bla*_PER-1_ gene. The prevalence of this gene in MDR *A*. *baumannii* clinical isolates has been extensively evaluated: Studies have reported a prevalence of 2% [56], 44% [189], 53% [102], and 64.2% [209]. Remarkably, in one study, out of 27 MDR *A*. *baumannii* clinical isolates, 44% had the *bla*_PER-1_ gene. These *bla*_PER-1_ positive isolates were recovered from the respiratory tract of patients. In contrast, *bla*_PER-1_ negative isolates were recovered from the bloodstream and were low biofilm formers [189], strongly suggesting a relationship between the expression of PER-1 and biofilm formation ability.

The capsule is a well-known virulence factor [210]. *epsA* and *ptk* are two genes involved in capsule biosynthesis in *A*. *baumannii*. *A*. *baumannii epsA* encodes for a putative polysaccharide export outer membrane protein (EpsA), whereas the *ptk* gene encodes for a putative protein tyrosine kinase (PTK) [165]. Both genes appear to be implicated in the biofilm formation of MDR *A*. *baumannii* isolates since a study involving 100 MDR *A*. *baumannii* clinical isolates demonstrated a 95% prevalence for both *ptk* and *epsA* [56], and all were strong biofilm formers. The presence of the capsule may contribute to a strong biofilm.

## Paths towards novel therapeutics

The exponential increase in antibiotic resistance along with the growing awareness of the deleterious impact of biofilms in medicine has led to a growing demand for new therapeutic alternatives. Several alternatives are currently under development, with the objective of combating AMR as well as the biofilms formation in *A*. *baumannii*. These include the development of new vaccines, the inhibition of quorum sensing, the use of nanoparticles and metal ions, natural products, antimicrobial peptides, and phage therapy.

### Vaccine development

The fight against AMR needs to be multifactorial, including stewardship of antimicrobials use, development of new therapeutics, and infection prevention. Vaccines targeting AMR and biofilm formation are being developed [211, 212]. A number of these aim at mounting immunity against structures of the cell wall that are involved, directly or indirectly, with biofilm formation. For *A*. *baumannii*, these include AbOmpA, Bap protein, and subunit proteins of the surface-exposed Csu pili such as CsuA/B [213–218]. AbOmpA appears to be the most relevant for future vaccine development against drug-resistant *A*. *baumannii*. Along with the fact that AbOmpA-antigen-based immunization demonstrates high protection and survival rates reaching 80% or higher [219, 220], AbOmpA is also highly conserved among *A*. *baumannii* clinical isolates, with prevalence reaching 99% [215, 216, 219]. A two-recombinant-pilus proteins vaccine targeting the CsuA/B protein plus a related fimbriae protein, FimA, was found to trigger protective immunity in mice against *A*. *baumannii* infection [217]. A Bap protein vaccine is similarly protective in murine models [218].

### Quorum sensing interference

Molecules that promote cell-to-cell communication, such as quorum sensing compounds (autoinducers), are attractive targets to reduce/inhibit biofilm formation. Quorum quenching inhibits bacterial cell communication. This can be accomplished by inhibiting the production or bacterial detection of these quorum sensing molecules or by their degradation [221]. Quorum quenching includes quorum quenching enzymes and quorum sensing inhibitors. Quorum quenching enzymes act in the extracellular environment and not intracellularly and hence will not select for resistant bacteria [221, 222].

Strategies of interference in quorum sensing are being developed in an attempt to prevent the formation of biofilms and the development of virulence factors as well as AMR in *A*. *baumannii* [136, 138, 223, 224]. As AHL lactonases destabilize quorum sensing in Gram-negative bacteria, these enzymes represent a promising approach to combat biofilm-associated infections and AMR [225, 226]. The utility of these enzymes as a therapeutic strategy against *A*. *baumannii* biofilms has been established using the clinical isolate *A*. *baumannii* S1 [223]. Similar results were observed with another lactonase enzyme with quorum quenching activity, MomL. This lactonase has the capability to degrade *A*. *baumannii* AHL and to dramatically reduce biofilm biomass while concurrently enhancing the susceptibility of the biofilm to antibiotics. Supplementation of antimicrobials with quorum quenching agents may offer synergistic benefits, as recently observed in *A*. *baumannii* biofilms exposed to tobramycin supplemented with MomL [224]. More research is warranted using this approach *in vivo* [224].

### Nanoparticles and metal ions

Various metal nanoparticles, particularly silver, are the topic of intense research activity in view of their antimicrobial properties [227]. The antimicrobial properties of metal nanoparticles have been evaluated both in planktonic bacterial cells and biofilms of different Gram-positive and Gram-negative bacterial species, including *A*. *baumannii*, demonstrating inhibition of biofilm formation and increased antimicrobial sensitivity [228–233].

A number of studies have evaluated the benefits of combining nanoparticles with other compounds to inhibit the formation of susceptible and MDR *A*. *baumannii* biofilms, some with promising outcomes. These findings are summarized in Table 3.

**Table 3:** Examples of studies that demonstrated the protective effects of nanoparticles alone and in combination with other compounds against biofilm formation in susceptible and MDR *A*. *baumannii*

Bacteria	Nanoparticles used	References
• Two hundred MDR wound infection clinical isolates of *A*. *baumannii*	Silver nanoparticles	[230]
• *A*. *baumannii* ATCC 19606 • One colistin-susceptible *A*. *baumannii* clinical isolate • Three colistin-resistant *A*. *baumannii* isolates	Colistin-loaded human albumin nanoparticles	[234]
• Clinical Carbapenem-resistant *A*. *baumannii* isolates	Silver nanocomposites	[235]
• MDR and non-MDR *A*. *baumannii*	Biosynthesized silver nanoparticles from *Galaxaura rugosa*	[236]
• Ten *A*. *baumannii* clinical isolates	Iron oxide nanoparticles	[237]

Silver nanoparticles inhibit DNA synthesis and induce apoptosis-like in MDR *A*. *baumannii* in a concentration-dependent fashion [238]. Silver nanoparticles derived from silver salts can inhibit biofilm formation of MDR *A*. *baumannii* strains, at least in part by inhibiting the expression of biofilm-related genes, such as *csuA/B*, *abompA*, and *bap* [230].

Antimicrobial and antibiofilm effects of metal ions solutions have been studied in both Gram-positive and Gram-negative bacteria [239, 240]. Antimicrobial effects have been reported against both planktonic and biofilm *A*. *baumannii* upon exposure to single and to combinations of metal ions solutions [239]. The antibacterial properties of metal ions are mediated by a variety of mechanisms including interference with bacterial cell membranes, generation of reactive oxygen species (ROS), protein destabilization, and DNA injury [241]. However, both chromosomal and plasmid-mediated resistance of bacteria to silver have been reported, as well as a co-selection with the use of antibiotics when the resistance genes rely on the same genetic platform, such as a plasmid-mediated silver efflux system, the Sil system [242]. More research is warranted to develop effective metal nanoparticle-based strategies to fight biofilm formation and AMR in MDR *A*. *baumannii*.

### Natural products

A few natural products under study have also demonstrated a high antibacterial and antibiofilm activity against MDR *A*. *baumannii* clinical isolates. These include cinnamaldehyde [243], essential oils [244, 245], and polyphenolic compounds [246]. Cinnamaldehyde has exhibited potent antibacterial and antibiofilm activity against carbapenem-resistant *A*. *baumannii* clinical isolates. The minimum bactericidal concentration of cinnamaldehyde on strong biofilm formers was 1.75 mg/mL. At ½ of the MIC, cinnamaldehyde inhibited biofilm formation by approximately 72% [243]. Essential oils can also achieve high antibacterial and antibiofilm activities against MDR *A*. *baumannii* clinical isolates. In nanoemulsion or as a pure compound, *Thymus daenensis* oils have achieved MICs of 45 μg/mL and 87.5 μg/mL, respectively. At ½ of the MIC, emulsions achieved 56.43% of inhibition of MDR *A*. *baumannii* clinical isolates biofilms [245]. Polyphenolic compounds have also been studied, presenting significant results, such as lower MICs and biofilm inhibition that can reach 90%, depending on the compound and isolate [246]. More research is warranted to identify the potential use of natural products and their mode of action in the dual control of biofilm formation and AMR in *A*. *baumannii*.

### Antimicrobial peptides

The study of antimicrobial peptides has enabled the identification of multiple compounds with relevant antibacterial and antibiofilm activity against MDR *A*. *baumannii* clinical isolates [247–249]. Magainin 2, isolated from the frog *Xenopus laevis* [247], and octominin, obtained from *Octopus minor* [248], have significant antibacterial and antibiofilm activity against MDR *A*. *baumannii* clinical isolates. At 4 μM, magainin 2 promoted biofilm inhibition in the resistant strains and a biofilm reduction of 66.2% at 256 μM. Octominin at 5 μg/mL promoted a biofilm inhibition of 61.59% and a biofilm reduction of 35.62%. Both magainin 2 and octominin were demonstrated to affect the *A*. *baumannii* cell membrane. Magainin 2 destabilizes both *A*. *baumannii* inner and outer membranes, whereas octominin permeabilizes the *A*. *baumannii* cell membrane, thus leading to bacterial cell death. Research needs to further characterize modes of action as well as identify potential off-target effects in infected patients.

### Phage therapy

Bacteriophages (also known as phages) are viruses that infect bacteria and, thus, have a promising potential to be used as an antibacterial therapy [250, 251]. The application of phage therapy as an antibacterial and antibiofilm strategy in MDR *A*. *baumannii* clinical isolates has yielded positive results, even in combination with other phages (cocktails) or antibiotics [251, 252].

Studies have assessed the antibacterial and antibiofilm activity of the phages AB7-IBB1 [253] and AB7-IBB2 [254] in MDR *A*. *baumannii* clinical isolates. With a multiplicity of infection (MOI) of 0.1 applied in 10^8^ CFU/well, the phage AB7-IBB1 inhibited biofilm formation on both abiotic and biotic surfaces in 40% and 50%, respectively. It also promoted more than 35% biofilm removal with a MOI of 10 in 10^6^ CFU/well. Similarly, phage AB7-IBB2 at a MOI of 0.1 in 10^8^ CFU/well, inhibited biofilm formation and disrupted preformed biofilms on abiotic surfaces in approximately 40%. Moreover, phages AB7-IBB1 and AB7-IBB2 were able to inhibit *A*. *baumannii* growth up to 46% and 70% with a MOI of 0.1 in 10^8^ CFU/well, respectively.

Phage therapy has proven to be an effective therapeutic option against MDR *A*. *baumannii* clinical isolates. However, *A*. *baumannii* can develop phage resistance in a very short period of time. Phage resistance in *A*. *baumannii* is related to mutations in *k* locus genes that culminate in relevant alterations on the capsule; the principal factor in with phage depends on adsorption [255, 256].

## Conclusion

The combination of AMR and biofilm formation makes *A*. *baumannii* a formidable enemy in healthcare settings, where it can cause a wide range of infections, including pneumonia, bloodstream, wound infections, and urinary tract infections. Resistance to last-resort antibiotics such as carbapenems and colistin has already been reported, allowing such strains to cause pan-drug-resistant infections that are presently impossible to eradicate. To address this global health threat, there is a need for improved surveillance, infection control measures, and the development of new antimicrobial agents and treatment strategies. *A*. *baumannii* biofilms provide a protective environment for the bacteria and further enhance their resistance to antimicrobial agents. Biofilms are also associated with the persistence of infections and the development of chronic diseases. A better understanding of the mechanisms that co-regulate biofilm formation and AMR will help identify new therapeutic targets. Key genes that are involved in biofilm formation, for example, the quorum sensing system AbaI/AbaR and the TCS BfmRS, are also implicated in the development of AMR. Many MDR clinical isolates contain genes like *abompA* and *csuE*, which are essential for biofilm genesis. New therapeutic strategies concurrently targeting these two phenomena include quorum sensing interference, developments of vaccines mostly targeting AbOmpA, the use of nanoparticles and metal ions, natural products, antimicrobial peptides, and phage therapy. All have shown promising results. However, the use of silver nanoparticles appears to come with a risk of *A*. *baumannii* developing plasmid-mediated resistance, highlighting the critical need for more research. Combined approaches that may confer synergistic benefits offer intriguing avenues towards new, effective, and safe therapies.
